# Global commercialization and research of veterinary vaccines against *Pasteurella multocida*: 2015–2022 technological surveillance

**DOI:** 10.14202/vetworld.2023.946-956

**Published:** 2023-05-09

**Authors:** Aníbal Domínguez-Odio, Daniel Leonardo Cala Delgado

**Affiliations:** 1 Dirección de Ciencia e Innovación. Grupo Empresarial LABIOFAM. Avenida Independencia km 16½, Boyeros, La Habana, Cuba; 2 Animal Science Research Group, Universidad Cooperativa de Colombia, Sede Bucaramanga, Carrera 33 N°, 30ª-05 (4.162,49 km) 68000, Bucaramanga, Colombia

**Keywords:** adjuvant, *Pasteurella* *multocida*, strain, technological surveillance, vaccine

## Abstract

**Background and Aim:**

*Pasteurella multocida* can infect a multitude of wild and domesticated animals, bacterial vaccines have become a crucial tool in combating antimicrobial resistance (AMR) in animal production. The study aimed to evaluate the current status and scientific trends related to veterinary vaccines against *Pasteurella multocida* during the 2015–2022 period.

**Material and Methods:**

The characteristics of globally marketed vaccines were investigated based on the official websites of 22 pharmaceutical companies. VOSviewer^®^ 1.6.18 was used to visualize networks of coauthorship and cooccurrence of keywords from papers published in English and available in Scopus.

**Results:**

Current commercial vaccines are mostly inactivated (81.7%), adjuvanted in aluminum hydroxide (57.8%), and designed to immunize cattle (33.0%). Investigational vaccines prioritize the inclusion of attenuated strains, peptide fragments, recombinant proteins, DNA as antigens, aluminum compounds as adjuvants and poultry as the target species.

**Conclusion:**

Despite advances in genetic engineering and biotechnology, there will be no changes in the commercial dominance of inactivated and aluminum hydroxide-adjuvanted vaccines in the short term (3–5 years). The future prospects for bacterial vaccines in animal production are promising, with advancements in vaccine formulation and genetic engineering, they have the potential to improve the sustainability of the industry. It is necessary to continue with the studies to improve the efficacy of the vaccines and their availability.

## Introduction

*Pasteurella multocida* is a zoonotic, opportunistic, fatal, highly contagious, and heterogeneous pathogenic agent, in terms of subspecies (*P. multocida* sub spp. multocida, gallicida, septica, and tigris), capsular serogroups (A, B, D, E, and F), and somatic lipopolysaccharides [1]. It has been reported that it can infect a wide spectrum of animals and cause mass mortality events, reproductive disorders, and significant losses in the production of milk, meat, and eggs [2, 3]. Another important characteristic of this pathogen is its relative predilection for specific hosts, despite the absence of any gene linking it to a specific animal species. International studies report high prevalence rates of serogroups A, B, and E in cattle and buffaloes, while infections in pigs are usually dominated by serogroups A and D. On the other hand, birds are affected mainly by serogroup A [4–6].

It is known that the virulence of these bacteria is complex and variable and depends on the strain, susceptible animals and contact conditions between both of these factors [1]. Several studies have shown that certain genes contribute to infection success [7] and encode the biosynthesis of key structural and metabolic molecules [8–10] required for adhesion, iron acquisition, colonization, immune response evasion, and survival in the host. Other researchers have identified important non-genetic factors involved in their pathogenesis; for instance, this microorganism is normally found in the oropharyngeal cavity [11–13], and immunological failures attributable to coinfections, environmental changes, or zootechnical stress are observed [14].

In veterinary medicine, chemotherapeutic control of this pathogen is questionable, as it produces residues in food and is expensive, time-consuming, and sometimes ineffective [15]. Therapeutic failures in *P. multocida* are largely attributable to the resistance developed by many wild strains toward antibiotics [13, 16]. Several control and mitigation measures have been established to deal with this problem, including the use of probiotic microorganisms [17], immune modulation through herbal compounds [18], and vaccination [19]. Despite the available options, vaccines are heavily responsible for the effective, safe, and economical prevention of disease [20]. The social and health responsibility placed on these formulations promotes multiple international investigations for their constant improvement and the creation of multidisciplinary strategic alliances to achieve this goal in the shortest possible time [21–24]. This scientific dynamism demands periodic monitoring by the veterinary biopharmaceutical industry to identify possible technological changes in advance, take advantage of new opportunities, and make adaptive decisions to the new scenario. These are important aspects for maintaining companies’ presence in the market and expanding their competitive advantage.

Based on these facts, this study aimed to evaluate the current status and scientific trends associated with veterinary vaccines against *P. multocida* during the 2015–2022 period.

## Materials and Methods

### Ethical approval

The approval from the Institutional Animal Ethics Committee to carry out this study was not required as no invasive technique was used.

###  Study period and location

An observational, descriptive, and retrospective study was performed from January to March 2022. General information on commercial vaccines against *P. multocida* was obtained from the official and public websites of 22 pharmaceutical companies having extensive presence in the market (Table-1). The bibliometric analysis included the scientific literature on *P. multocida*, strain, vaccine, and adjuvants published during the 2015–2025 period in English in the original paper modality, refereed by peers, indexed, and available on the Scopus platform.

**Table-1:** Pharmaceutical companies with experience in the development, production and marketing of veterinary vaccines against *P. multocida* included in the study.

Pharmaceutical company	Country	Website
BioChemiq	Argentina	www.biochemiq.com
Biogénesis Bagó S. A	Argentina	www.biogenesisbago.com
Bioveta Ltd	Czech Republic	www.bioveta.eu
BioZoo	Mexico	www.biozoo.com
Boehringer Ingelheim	Germany	www.boehringer-ingelheim.com
CEVA Santé Animale	France	www.ceva.com
ELANCO	USA	www.elanco.com
Finmark Laboratorios S. A	Colombia	www.finlab.com.co
Farvet	Peru	www.farvet.com
Hester Biosciences Ltd	India	www.hester.in
Inst. Vet. Res. Dev. Vietnam*	Vietnam	www.vinoda.vn
Instituto Rosenbusch S. A	Argentina	www.rosenbusch.com
James Brown Farma C.A	Ecuador	www.jamesbrownpharma.com
Kenya Vet. Vac. Prod. Inst.**	Kenya	www.kevevapi.or.ke
Laboratorios HIPRA, S.A.	Spain	www.hipra.com
Lavet	Mexico	www.grupolavet.com
Microsules	Uruguay	www.laboratoriosmicrosules.com
MSD Animal Health, S.L.	USA	www.msd-animal-health.com
Stavropol Biofabrika	Russia	www.en.stavbio.ru
Vecol	Colombia	www.vecol.com.co
Vet. Serum Vac. Res. Inst.***	Egypt	www.vsvri-eg.com
Zoetis	USA	www.zoetis.com

* Institute for Veterinary Research and Development of Vietnam,

** Kenya Veterinary Vaccines Production Institute,

*** Veterinary Serum and Vaccine Research Institute

### Search strategy

Common criteria were established for the 22 manufacturers to group and compare the different commercial vaccines used to immunize domestic animals against *P. multocida*. The collected and filtered information was about the production technology used (traditional or modern), capsular serogroups of the vaccine strain (A, B, D, E, and F), type of adjuvants (aluminum salts, emulsion, oily, and natural), target animal species (cattle, sheep-goats, pigs, horses, poultry, and rabbits), and types of formulation (monovalent or polyvalent and viral or bacterial).

In the bibliometric analysis, the fields “title,” “abstract,” and “keywords” were used to identify the relevant publications associated with the subject of veterinary vaccines against *P. multocida*. The specific terms such as *P. multocida*, strain, vaccine, adjuvant, bovine, sheep-goat, porcine, equine, avian, and rabbit were combined with their grammatical variants frequently used in the English language (Table-2). The references obtained were exported from Scopus to the VOSviewer version 1.6.18 software (Centre for Science and Technology Studies of Leiden University, Netherlands) to conduct a coauthorship analysis by country and cooccurrence.

**Table-2:** Keywords and grammatical variants used to identify relevant publications in Scopus.

Keywords	Grammatical variants
*Pasteurella multocida*	Pasteurellosis, hemorrhagic septicemia, fowl cholera, pneumonic pasteurellosis, atrophic rhinitis, and snuffle
Strain	Vaccine strain, reference strain, wild strain, field strain, mutant strain, and avirulent strain
Vaccine	Bacterin, traditional vaccine, live attenuated vaccine, killed vaccine, inactivated vaccine, recombinant vaccine, DNA vaccine, and subunit vaccine
Adjuvant	Alum, aluminum hydroxide, aluminum salts, mineral adjuvants, emulsions, oil emulsions, oil adjuvants, natural adjuvant, saponin adjuvant, and polymers
Bovine	Cattle, cow, calf, calves, ruminant, domestic ruminants, livestock, farm animals, food animals, and farm animals
Sheep-goat	Small ruminant, ovine, caprine, goat, goatish, livestock, farm animals, food animals, and farm animals
Porcine	Pig, hog, swine, piglets, food animals, and farm animals
Equine	Horse and farm animals
Avian	Poultry, chicken, bird, turkey, duck, and food animals

The references obtained were subjected to a metadata normalization process to eliminate erroneous or duplicate documents through the use of EndNote. After carrying out this procedure, a manual revision of the content was carried out and the final sample was exported to the VOSviewer version 1.6.18 software for the visualization of network-based maps.

### Analysis and representation of data

The descriptive statistics of the indicators linked to the production of commercial veterinary vaccines against *P. multocida* marketed during 2022 were carried out using the Microsoft Excel 2019 program. Then, the data were summarized in figures where the frequencies and percentages were presented. The metric analysis of the scientific production was achieved by creating network maps for the collaboration between countries and the cooccurrence of keywords, with a minimum of five matches.

## Results and Discussion

### Commercial vaccines against *P. multocida*

**Figure-1:**
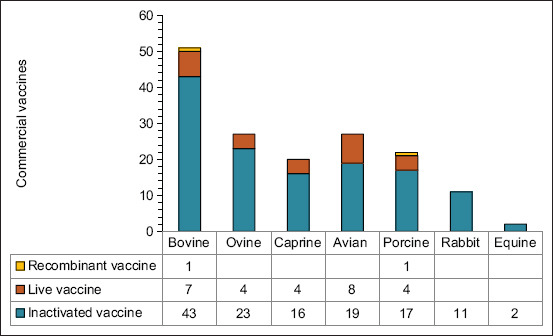
Distribution of commercial veterinary vaccines against *Pasteurella multocida* according to animal species during 2022.

Detailed analysis of the information provided by the manufacturers on their official and public websites indicated that inactivated vaccines most frequently appeared on the market (81.9%, 131/160), compared with the live attenuated and recombinant ones (Figure-1). Similarly, the distribution of vaccines according to animal species showed that the largest market share belongs to ruminants, at 61.2% (98/160); whereas the remaining 38.8% (62/160) is represented by poultry, pigs, rabbits, and horses to a lesser extent (Figure-1). Bovine could be identified as the leading species in the market, as 31.9% (51/160) of all obtainable vaccines were available, of which 84.3% (43/51) were inactivated.

Explaining this behavior implies recognizing that inactivated vaccines are effective and have an excellent cost-benefit ratio, despite generating short-lived immunity, and insufficient cross-protection [25–28]. Another favorable aspect of the industrial production of these formulations are, the fewer regulatory restrictions than those for other types of vaccines. The manufacturer is not required to have in-depth knowledge of the molecular structure of these vaccines [29], which accelerates their development and reduces the time required for obtaining the official marketing authorization.

The possibility that some countries may have to manufacture their own vaccines from local circulating strains is another factor that should not be ignored when analyzing the commercial hegemony of inactivated vaccines [10, 30–33]. Autogenous vaccines, in general terms, are a solution for the protection of certain herds against various autochthonous pathogenic strains [30, 31] or for the local elimination of strains carrying genes for antibiotic resistance [34]. In a practical sense, it also means self-sufficiency, technological sovereignty, low prices per dose, and the possibility of exporting to neighboring regions.

The obvious health, technological, practical, and economic advantages of inactivated autogenous *P. multocida* vaccines seem to outweigh the risks involved in their use. The greatest risk is related to the occurrence of heterologous pathogenic strains in vaccinated herds and against which the vaccine is ineffective [35]. Antigenic drift and variable safety are other disadvantages that were identified, the latter being specific to the production technology employed. The inactivation of bacterial cells generates many antigenic structures, which compete to stimulate the host’s immune system and may induce suboptimal levels of protection and undesirable adverse effects [29].

**Figure-2:**
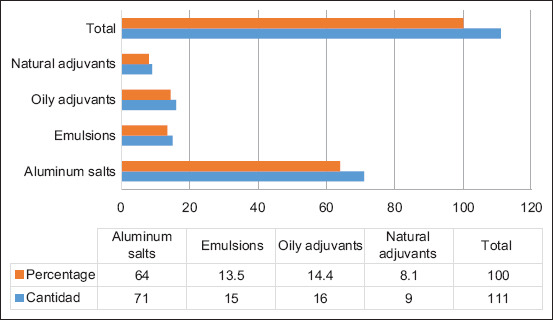
Distribution of adjuvants used in commercial veterinary vaccines against *Pasteurella multocida* according to their origin during 2022.

Regarding the official use of adjuvants, it was found that not all inactivated commercial formulations declared the type of immunopotentiator used even though its use was indicated. Of the vaccines studied, 15.3% (20/131) did not provide this important information; whereas, 84.7% (111/131) met this requirement. Under these particular conditions, the distribution of adjuvants according to origin showed (Figure-2) the predominance of aluminum salts (64.0%, 71/111), followed by oily adjuvants (14.4%, 16/111), emulsions (13.5%, 15/111), and finally, adjuvants of natural origin (8.1%, 9/111).

Although data bias distorts the actual participation of the different adjuvants in commercial vaccines, we confirmed a limited variety of compounds used for commercial purposes and the hegemony of aluminum salts (aluminum hydroxide). The prevalence of this compound was expected and was confirmed by a recent study on *P. multocida* vaccines worldwide [27]. The popularity of this compound in the veterinary pharmaceutical industry is attributed to its long history of use, known structure, demonstrated stability, easy preparation, simplicity of purchase, and low manufacturing costs [36]. The capacity of aluminum hydroxide to internalize the antigen without modifying its structure and then releasing it for prolonged periods, along with its proven ability to stimulate the production of cytokines and polymorphonuclear cells, undoubtedly contributes to its widespread acceptance [37]. Formulations that include it, therefore, have shorter development times, lower registration fees, and rapid return on investment [38].

Regarding the use of vaccine strains, we observed similar difficulties as those for adjuvants. Manufacturers prioritized disclosing the capsular type, to the detriment of its commercial name, perhaps because they were isolated locally. Predominance of the Type A serogroup was remarkable in this regard. The data collected showed that 83.1% (133/160) of the commercial vaccines include it exclusively or combine it with types B and D. This commercial strategy is based on the high prevalence of these serogroups in the field [6, 33, 39], particularly the A serogroup [40, 41] and due to the absence of cross-protection between them [42].

Vaccines against *P. multocida* had the additional characteristic of being formulated with various antigens (Table-3). Although the composition of the resulting polyvalent vaccines was heterogeneous, it was found that bacterial antigens (80.6%, 25/31) were more dominant than viral antigens (19.4%, 6/31). *Clostridium* and *Salmonella* were of the greatest industrial interest, and both represented 48.0% (12/25) of all bacterial antigens available on the market. Cattle once again received the most attention, and 74.2% (23/31) of the antigens declared by pharmaceutical companies were used to immunize them.

**Table-3:** Bacterial and viral antigens used together with *P. multocida* in commercial polyvalent vaccines distributed according to animal species.

Infectious agent	Animal species
*A. pleuropneumoniae*	Porcine
*A. paragallinarum*	Avian
*B. bronchiseptica*	Porcine
*C. chauvoei*	Bovine, ovine-caprine, equine
*C. haemolitycum*	Bovine, ovine
*C. novyi*	Bovine
*C. perfringens*	Bovine, ovine-caprine
*C. septicum*	Bovine, ovine-caprine, equine
*C. sordellii*	Bovine
*C. pyogenes*	Bovine
*E. coli*	Bovine, ovine, avian, porcine
*G. anatis*	Avian
*H. somnus*	Bovine
*H. somni*	Bovine, ovine-caprine
*L. interrogans*	Bovine
*M. haemolytica*	Bovine, ovine-caprine, rabbits, equine, porcine
*M. bovis*	Bovine
*M. bovoculi*	Bovine
*S. enteritidis*	Bovine, ovine, porcine
*S. cholera­suis*	Porcine, bovine, ovine, equine
*S. dublin*	Bovine, ovine, equine, porcine
*S. gallinarum*	Avian
*S. newport*	Bovine, ovine, equine, porcine
*S.* Typhimurium	Bovine, avian, porcine
*S. faecalis*	Porcine
Classical swine fever	Porcine
Bovine herpesvirus type 1	Bovine
Bovine parainfluenza type 3	Bovine
Newcastle virus	Avian
Bovine viral diarrhea virus	Bovine
Bovine respiratory syncytial virus	Bovine

*P. multocida*=*Pasteurella multocida*, *A. pleuropneumoniae*=*Actinobacillus pleuropneumoniae*, *A. paragallinarum*=*Avibacterium paragallinarum*, *B. bronchiseptica*=*Bordetella bronchiseptica*, *C. chauvoei*=*Clostridium chauvoei*, *C. haemolitycum*=*Clostridium haemolitycum*, *C. novyi*=*Clostridium novyi*, *C. perfringens*=*Clostridium perfringens*, *C. septicum*=*Clostridium septicum*, *C. sordellii*=*Clostridium sordellii*, *C. pyogenes*=*Corynebacterium pyogenes*, *E. coli*=*Escherichia coli*, *G. anatis*=*Gallibacterium anatis*, *H. somnus*=*Haemophilus somnus*, *H. somni*=*Histophilus somni*, *L. interrogans*=*Leptospira interrogans*, *M. haemolytica*=*Mannheimia haemolytica*, *M. bovis*=*Moraxella bovis*, *M. bovoculi*=*Moraxella bovoculi*, *S. enteritidis*=*Salmonella enteritidis*, *S. cholera­suis*=*Salmonella cholera­suis*, *S. dublin*=*Salmonella dublin*, *S. gallinarum*=*Salmonella gallinarum*, *S. newport*=*Salmonella newport*, *S. typhimurium*=*Salmonella* Typhimurium, *S. faecalis*=*Streptococcus faecalis*

The combination of multiple antigens in the same formulation promotes the prevention of a greater number of diseases per dose, rapid compliance with the vaccination schedule, and increased immunization coverage. In practical terms, it reduces the costs of application and transport and storage of the biological, as well as decreases the stress of the cattle due to less handling. Its major limitations, however, are possible interference between multiple antigens, uncertainty regarding the optimal time of administration and adverse reactions that are difficult to attribute to a specific component [42, 43].

The heterogeneity of the infectious agents combined with *P. multocida* in commercial polyvalent vaccines is consistent with the health priorities of the different breeding systems established for productive species. Mixing species with different susceptibilities, overcrowding and stressful factors during rearing favor the spread of primary infections in the herd. The immunological failure is generated and the histological lesions initially caused by these agents provide the ideal setting for *P. multocida* to opportunistically invade different host organs and worsen clinical symptoms. The productive damage initiated by the primary pathogen is exacerbated under these conditions [14, 44, 45], which is an aspect that can be made more complex by the presence of resistant antibiotic strains [16]. Therefore, it is not surprising that in multi-etiological infections where *P. multocida* is present together with *Avibacterium paragallinarum*, *Bordetella bronchiseptica*, *Clostridium* spp., *Escherichia* *coli*, *Mannheimia* *haemolytica*, and *Salmonella* spp., high mortality rates are reported [45–47] and, consequently, serious economic losses.

The hegemony of cattle in commercial vaccination against *P. multocida* was expected. This species plays an important role in the global food production for human consumption [48] and has an increased susceptibility to the pathogen and one of the highest mortality rates due to this cause [27]. In this regard, literature reports frequent coinfections where *M. haemolytica*, *Histophilus somni*, *Haemophilus somnus*, bovine respiratory syncytial virus, bovine herpesvirus Type 1, bovine viral diarrhea virus, bovine parainfluenza Type 3, and *P. multocida* participate synergistically [14, 44, 49–51].

### Scientific trends in vaccines against *P. multocida*

**Figure-3:**
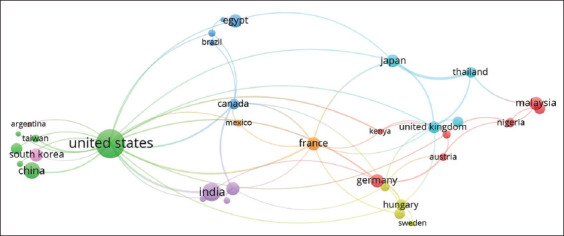
Bibliographic map that shows the coauthorship and strength of the links established between countries in the field of investigation on veterinary vaccines against *Pasteurella multocida* during the 2015–2022 period. Each color represents a different domain; the size of each node is proportional to the frequency of occurrence of joint publications between countries/region, whereas the lines represent the interaction between countries/region and the frequency of coauthorship (the smaller the distance, the greater the frequency of coauthorship).

Figure-3 shows the extensive international network of coauthors linked to publications on vaccines against *P. multocida*. A total of 65 countries were identified as being part of the coauthorship network, of which 21 were highlighted for the representativeness of their actions, with greater than five matches in the 2015–2022 period. It was observed that four countries grouped the 37.90% (246/649) of all the articles written in English contained in Scopus, where the United States with 142 publications, leads the ranking (21.9%), followed by India (48/649, 7.4 %), China (36/649, 5.5%), and Egypt (20/649, 3.1%).

The eight clusters, visualized in Figure-3, show a high interrelationship between the countries in terms of research on vaccines against *P. multocida*. The United States, France, Germany, the United Kingdom, and Japan, by showing investigative alliances with China, Argentina, India, Mexico, Brazil, Kenya, Egypt, and Thailand, showed that they all face a common enemy, regardless of the degree of development of their respective economies.

The results of Almoheer *et al*. [27] shows similarity with other studies conducted during 2005–2019, which may partly be because of the global distribution of the pathogen, its ability to cause mass mortality events, and the diversity of affected animals [4–6, 52–58]. From the economic standpoint, it reflects the enormous pressure to minimize losses while raising productive animals and the need to meet the sustained increase in the global demand for animal protein [48]. Other additional motivations that may be stimulating this fact are, increasing demands in terms of food safety [59], high zoonotic risk, emerging epidemiological behavior of the disease, and the urgency to establish control plans adapted to the health realities of each country [12, 19].

**Figure-4:**
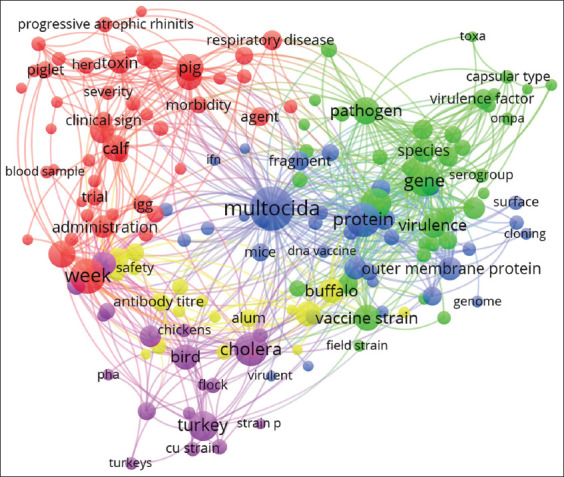
Bibliographic map showing the cooccurrence of keywords and the strength of the links established between the thematic areas dedicated to the investigation of veterinary vaccines against *Pasteurella multocida* during the 2015–2022 period. Each color represents a different domain; the size of each node is proportional to its frequency of occurrence, whereas the lines represent the interaction between terms and the frequency of cooccurrence in each study (the smaller the distance, the higher the frequency of cooccurrence).

Figure-4 shows the intense and complex knowledge network generated by research on veterinary vaccines against *P. multocida* during the 2015–2022 period. The distribution of the 649 publications found in Scopus indicated that the greatest scientific production was concentrated towards the search for new options to immunize poultry (44.1%), followed by pigs (24.4%), ruminants (cattle, buffalo, and sheep or goats) at 15.7% and rabbits (14.9%). In addition, five fundamental thematic domains were identified with a close relationship to each other: strains (green region with 39 nodes), immunogenicity (blue region with 28 nodes), clinical evaluations in pigs-ruminant (red region with 46 nodes), clinical evaluations in birds (purple region with 39 nodes), and adjuvants (yellow region with 18 nodes).

**Figure-5:**
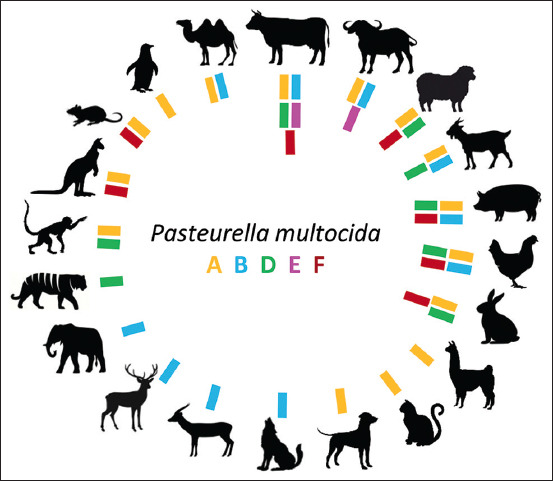
International reports of *Pasteurella multocida* grouped according to capsular serogroup and animal species. “Poultry” represents the reports made for chickens, turkeys, and ducks.

The main lines of research of the thematic domain strains (green region) were pathogenicity genes, design of genetic variants, and molecular epidemiology. Specifically, the virulence genes *ompA*, *pfhA*, *ptfA*, *tbpA*, *hgbB*, *toxA*, *oma*87, soda, and *nanB* and the mutations Δ*fur*, Δ*crp*, Δ*pcgD*, Δ*hptE*, and Δ*gdhA* showed the greatest scientific interest. The scientific production on the characterization of circulating pathogenic strains was high, possibly favored by the existing global genetic diversity and the large number of species affected (Figure-5). Serogroups A, B, and D received the greatest attention from science, associated with their frequent isolation during outbreaks in ruminants (cattle, buffaloes, and sheep or goats), pigs, and poultry.

The constant genetic monitoring of circulating strains carried out by science was vital for the pharmaceutical industry, since it allowed knowing the antigenic spectrum per host [39, 60] and explaining possible failures observed in vaccines made with reference vaccine strains [61]. However, its greatest utility is directed toward two very important areas that are not mutually exclusive: First, to identify candidate strains to develop new vaccines and second, to update existing vaccination strategies to adjust them to the epidemiological realities of each region [5, 30, 62].

The thematic domains of immunogenicity (blue region) and *in vivo* evaluation in pigs, ruminants, and poultry (red and purple regions) formed a very dense network. All these thematic areas were very close to each other and showed a high number of links, which accounts for the great scientific interest in finding specific immunogens for each productive species or region.

The results obtained from the search equation used in this study indicated that proteins are the most widely researched antigenic structures internationally and, therefore, the most influential in developing new vaccines against *P. multocida*. In general, the scientific literature specially emphasized the importance of obtaining and purifying a wide range of protein-derived molecules: Peptide fragments, surface lipoproteins (*PlpE* and *PlpB*), toxins, Type 4 fimbriae (*ptfA*), and recombinant outer membrane proteins (*rOmpH*, *rOmp16*, and *rTbpA*). The origin of these antigenic structures was equally diverse, ranging from reference vaccine strains (X73, CVCC446, M-1404, P-1059, P-470, P-1662, P52, P-61, and LZ-PM) to field isolates originating mostly from diseased turkeys, poultry, pigs, buffalo, and cattle.

On the other hand, the adjuvants (yellow region) had low visualization in the cooccurrence analysis compared to the other clusters. Despite only a few studies identified, it was observed that aluminum salts and their respective safety–effectiveness studies showed the highest frequency. The buffalo species had the greatest thematic weight and, therefore, the greatest number of publications.

### *In vivo* evaluation of new vaccines against *P. multocida*

Developing new vaccines against *P. multocida* is a challenging task, as is the case in many veterinary infectious diseases [63]. For years, overcoming the indicators of safety, efficacy, and duration of immunity achieved with traditional vaccines has been highly desirable [42]. However, more and better knowledge about pathogenesis, virulence factors, target populations, and routes of antigen administration, among other aspects, are required to develop better vaccines.

The hope of obtaining live vaccines with added value, including the possibility of self-transmission from a vaccinated animal to an unvaccinated animal makes science pay special attention to the design of mutant strains. However, the studies conducted using some candidates (Δ*fur*, Δ*crp*, Δ*pcgD* and Δ*hptE* among others) in experimental models and different routes of administration (oral, intranasal, and intramuscular) did not produce the expected results or were unfavorable. Although the new knowledge about the bacterial genome was used as a platform to manipulate the molecular regulators of *P. multocida*, it was not sufficient to create a strain with successful genetic attenuation. In all cases, the virulence and severity of the lesion is reduced, but the mutant strains retain the same limitations as live vaccines: Low homologous protection (60%–62%), risks of possible reversion to virulent character, and dependence on the cold chain. Some results in this sense confirm that a deletion is not enough to achieve a highly safe and efficient vaccine. In the near future, it will be necessary to continue searching for the most suitable combination, in order to evaluate the response of the immune system in different hosts later [64–67].

The results of the preclinical and clinical immune response assessments obtained with the different vaccine candidates (subunit and recombinant) were generally controversial and sometimes did not exceed the effectiveness of conventional vaccines. Even though these formulations were diverse from the molecular and antigenic standpoint, they are classified as safe and effective in general terms, thus reducing the onset of clinical symptoms of the disease in rodents, cattle, buffaloes, and birds. They induced high titers of interferon, IgA and IgG within weeks after intranasal or subcutaneous administration. However, similar to traditional vaccines (live and inactivated), these candidate vaccines need booster doses and fail to provide heterologous protective immunity in animals exposed to them, with many of these attempts requiring to optimize immunological indicators for assessing the efficacy, safety, and stability of the protein construct. In other cases, several issues must be resolved in the immediate future, including selecting better fragments of the original protein to use them individually or in fusion to achieve better immunogenic performance [68–75].

The DNA vaccines were also part of the new research options shown in the immunogenicity domain (blue), although they were less representative compared to protein vaccines. As a general strategy, this new preventive strategy included intramuscular or intranasal transfection of genes encoding protein antigens (*OmpH*, *OmpA*, and *ptfA*), the use of rodents as an experimental model and challenges with pathogenic strains of the capsular A serogroup.

The promising results published based on formulations with naked molecules and molecules encapsulated with nanoparticles show that the vaccine candidates are stable, safe, and induce lymphocyte proliferation, high antibody titers (IgA, IgG, and IgM), and gamma interferon. These results should be interpreted with caution because the protective effects of most of the DNA vaccines investigated during the 2015–2022 period are similar or inferior to those of traditional vaccines. In this regard, many open questions remain, as the protective efficacy of plasmids in many target species, the mechanism of action and the duration of immunity are unknown [76–79]. Moreover, its greatest challenge will be overcoming the numerous regulations that will govern the presumed application of these vaccines in domestic animals in the future.

Regarding the research activities on adjuvants, it can be stated that aluminum compounds attracted the greatest scientific interest during the evaluated period, including their use in the form of nanoparticles [80]. The remaining investigations available on this topic tried to demonstrate the safety and efficacy of several oily adjuvants [81] and emulsions [82, 83] in different animal species. They all attempted to overcome the strong Th2-type stimulation generated by aluminum hydroxide, which is a highly appreciated and desirable property for any adjuvant involved in combating extracellular pathogens, such as *P. multocida* [84].

The remaining *in vivo* studies in this field of research explored, to a lesser extent, the novel use of bacterial DNA [85] and natural compounds [86, 87]. Different compounds of plant and animal origin converged with varying degrees of experimental development in this last small group of substances. The biopolymer chitosan and the polysaccharide inulin can be mentioned among the most advanced compounds, which have a certain degree of application in recombinant subunits [65] and DNA vaccines [74, 84]. Despite these examples, it can be stated that their level of application is low, and it will take a long time to prove that they are stable, reproducible, robust, and scalable. Therefore, these compounds cannot be expected to be used in industrial production in the short term.

## Conclusion

Overall, although some formulations have shown promising results and clear potential, more research and larger-scale trials are required before the described experimentally developed vaccines can be commercialized. The absence of signs of change allows us to infer that, regarding *P. multocida*, there will be no significant modifications in the short term (3–5 years) that will affect the commercial domain of inactivated and adjuvanted vaccines with aluminum hydroxide. Industry and research will continue to focus on food animals.

## Authors’ Contributions

AD: Conceived the original idea. AD and DLCD: Acquisition, analysis, and interpretation of data, discussion, and prepared the manuscript. Both authors have read, reviewed, and approved the final manuscript.

## References

[1] Harper M, Boyce J.D, Adler B (2006). *Pasteurella multocida* pathogenesis:125 years after Pasteur. FEMS Microbiol. Lett.

[2] Clemmons E.A, Alfson K.J, Dutton J.W (2021). Transboundary animal diseases, an overview of 17 diseases with potential for global spread and serious consequences. Animals (Basel).

[3] Orynbayev M, Sultankulova K, Sansyzbay A, Rystayeva R, Shorayeva K, Namet A, Fereidouni S, Ilgekbayeva G, Barakbayev K, Kopeyev S, Kock R (2019). Biological characterization of *Pasteurella multocida* present in the Saiga population. BMC Microbiol.

[4] Shivachandra S.B, Viswas K.N, Kumar A.A (2011). A review of haemorrhagic septicaemia in catte and buffalo. Anim. Health Res.

[5] Pors S.E, Hansen M.S, Christensen H, Jensen H.E, Petersen A, Bisgaard M (2011). Genetic diversity and associated pathology of *Pasteurella multocida* isolated from porcine pneumonia. Vet. Microbiol.

[6] Peng Z, Liang W, Wang F, Xu Z, Xie Z, Lian Z (2018). Genetic and phylogenetic characteristics of *Pasteurella multocida* isolates from different host species. Front. Microbiol.

[7] Hurtado R, Maturrano L, Azevedo V, Aburjaile F (2020). Pathogenomics insights for understanding *Pasteurella multocida* adaptation. Int. J. Med. Microbiol.

[8] Peng Z, Wang X, Zhou R, Chen H, Wilson B.A, Wu B (2019). *Pasteurella multocida*:Genotypes and genomics. Microbiol. Mol. Biol. Rev.

[9] Guan L, Xue Y, Ding W, Zhao Z (2019). Biosynthesis and regulation mechanisms of the *Pasteurella multocida* capsule. Res. Vet. Sci.

[10] Cruz G.G, Lara L.L, Fresán M.U.A, de Oca Jiménez R.M, Fernández P (2010). Immunogenic evaluation and protection of *Pasteurella multocida* antigens isolated from clinical cases [Evaluación de la capacidad inmunogénica y protectora de antígenos de *Pasteurella multocida*, obtenidos de aislados de casos clínicos]. Vet. Méx.

[11] Tinmaz T, Çelik B, Halaç B, Bağcigil A (2021). Characterization of *Pasteurella multocida* isolates recovered from the oral flora of cats. Ank. Univ. Vet. Fak. Derg.

[12] Kutzer P, Szentiks C.A, Bock S, Fritsch G, Magyar T, Schulze C (2021). Re-emergence and spread of haemorrhagic septicaemia in Germany:The wolf as a vector?. Microorganisms.

[13] Rabana J, Ibrahim A, Ayuba M, Ibrahim U (2021). Prevalence and antimicrobial susceptibility profiles of *Pasteurella multocida* in village chickens (*Gallus gallus domesticus*) in Maiduguri, Borno State, Nigeria. Am. J. Biomed. Sci.

[14] Sudaryatma P.E, Nakamura K, Mekata H, Sekiguchi S, Kubo M, Kobayashi I, Okabayashi T (2018). Bovine respiratory syncytial virus infection enhances *Pasteurella multocida* adherence on respiratory epithelial cells. Vet. Microbiol.

[15] Martin N, Suarez N (2019). Inquietudes Respecto a la Administración de Antibióticos en la Medicina Veterinaria. Papeles UAN.

[16] Bourély C, Cazeau G, Jouy E, Haenni M, Madec J.Y, Jarrige N, Leblond A, Gay E (2019). Antimicrobial resistance of *Pasteurella multocida* isolated from diseased food-producing animals and pets. Vet. Microbiol.

[17] Reuben R, Sarkar S, Ibnat H, Setu M.A.A, Roy P, Jahid I (2022). Multi-strain probiotics upregulated anti-inflammatory properties and reduced *Pasteurella multocida* mortality in broilers. Int. J. Infect. Dis.

[18] Ayrle H, Mevissen M, Kaske M, Nathues H, Gruetzner N, Melzig M, Walkenhorst M (2016). Medicinal plants--prophylactic and therapeutic options for gastrointestinal and respiratory diseases in calves and piglets?A systematic review. BMC Vet. Res.

[19] Micoli F, Bagnoli F, Rappuoli R, Serruto D (2021). The role of vaccines in combatting antimicrobial resistance. Nat. Rev. Microbiol.

[20] Hoelzer K, Bielke L, Blake D.P, Cox E, Cutting S.M, Devriendt B, Van Immerseel F (2018). Vaccines as alternatives to antibiotics for food-producing animals. Part 2:New approaches and potential solutions. Vet. Res.

[21] Mostaan S, Ghasemzadeh A, Ehsani P, Sardari S, Shokrgozar M.A, Abolhassani M, Brujeni G (2021). *In silico* analysis of *Pasteurella multocida* PlpE protein epitopes as novel subunit vaccine candidates. Iran. Biomed. J.

[22] Aida V, Pliasas V. C, Neasham P. J, North J. F, McWhorter K. L, Glover S. R, Kyriakis C. S (2021). Novel vaccine technologies in veterinary medicine:a herald to human medicine vaccines. Front. Vet. Sci.

[23] Charleston B, Graham S (2018). Recent advances in veterinary applications of structural vaccinology. Curr. Opin. Virol.

[24] Nooraei S, Sarkar Lotfabadi A, Akbarzadehmoallemkolaei M, Rezaei N (2023). Immunogenicity of different types of adjuvants and nano-adjuvants in veterinary vaccines:a comprehensive review. Vaccines.

[25] Qureshi S, Saxena H.M (2014). Estimation of titers of antibody against *Pasteurella multocida* in cattle vaccinated with haemorrhagic septicemia alum precipitated vaccine. Vet. World.

[26] Guan L.J, Song J.J, Xue Y, Ai X, Liu Z.J, Si L.F, Li M.Y, Zhao Z.Q (2021). Immune protective efficacy of China's traditional inactivated and attenuated vaccines against the prevalent strains of *Pasteurella multocida* in mice. Vaccines (Basel).

[27] Almoheer R, Abd Wahid M.E, Zakaria H.A, Jonet M.A.B, Al-Shaibani M.M, Al-Gheethi A, Addis S.N.K (2022). Spatial, temporal, and demographic patterns in the prevalence of hemorrhagic septicemia in 41 countries in 2005–2019:A systematic analysis with special focus on the potential development of a new-generation vaccine. Vaccines (Basel).

[28] Belyaeva A, Kapustin A, Shastin P, Ivanov E, Laishevtsev A (2022). Development and testing of a vaccine against infectious atrophic rhinitis and pasteurellosis in pigs. BIO Web Conf.

[29] Heldens J.G.M, Patel J.R, Chanter N, Ten Thij G.J, Gravendijck M, Schijns V.E.J (2008). Veterinary vaccine development from an industrial perspective. Vet. J.

[30] Akhtar M, Rahman M.T, Ara M.S, Rahman M, Nazir K.N.H, Ahmed S, Rahman M (2016). Isolation of *Pasteurella multocida* from chickens, preparation of formalin killed fowl cholera vaccine, and determination of efficacy in experimental chickens. J. Adv. Vet. Anim. Res.

[31] Domínguez-Odio A, Acosta-Dueñas P, Oliva-López M, Rosales-Boch K (2021). Cuban vaccine against rabbit *Pasteurella multocida*:52 years of immunization [Vacuna cubana contra *Pasteurella multocida* cunícula:52 años de inmunización]. Rev. Salud Anim.

[32] Wubet W, Bitew M, Mamo G, Gelaye E, Tesfaw L, Sori H (2019). Evaluation of inactivated vaccine against fowl cholera developed from local isolates of *Pasteurella multocida* in Ethiopia. Afr. J. Microbiol. Res.

[33] Valadan M, Jabbari A.R, Niroumand M.T, Tahamtan Y, Bani H (2014). Isolation and identification of *Pasteurella multocida* from sheep and goat in Iran. Arch. Razi Inst.

[34] Barnes C, Rudenko O, Landos M, Dong H. T, Lusiastuti A, Phuoc L. H, Delamare-Deboutteville J (2022). Autogenous vaccination in aquaculture:A locally enabled solution towards reduction of the global antimicrobial resistance problem. Rev. Aquac..

[35] Omaleik L, Blackall P.J, Turni C (2020). Using genomics to understand inter- and intra-outbreak diversity of *Pasteurella multocida* isolates associated with fowl cholera in meat chickens. Microb. Genom.

[36] Akache B, Stark F.C, Agbayani G, Renner T.M, McCluskie M (2022). Adjuvants:Engineering protective immune responses in human and veterinary vaccines. Methods Mol. Biol.

[37] Ghimire T.R (2015). The mechanisms of action of vaccines containing aluminum adjuvants:An *in vitro* vs. *in vivo* paradigm. SpringerPlus.

[38] Del Giudice G, Rappuoli R, Didierlaurent A.M (2018). Correlates of adjuvanticity:A review on adjuvants in licensed vaccines. Semin. Immunol.

[39] Vu-Khac H, Trinh T.T.H, Nguyen T.T.G, Nguyen X.T, Nguyen T.T (2020). Prevalence of virulence factor, antibiotic resistance, and serotype genes of *Pasteurella multocida* strains isolated from pigs in Vietnam. Vet. World.

[40] Xiao J, Li Y, Hu Z, Zhang Y, Chang Y.F, Zhou Q, Xie Q (2021). Characterization of *Pasteurella multocida* isolated from ducks in China from 2017 to 2019. Microb. Pathog.

[41] Mirtneh Y.A, Vemulapati B.M, Takele A, Martha Y, Teferi D, Alebachew B, Esayas G (2022). Phenotypic and molecular characterization of the capsular serotypes of *Pasteurella multocida* isolated from pneumonic cases of cattle in Ethiopia. Agric. Sci. Digest.

[42] Mostaan S, Ghasemzadeh A, Sardari S, Shokrgozar M.A, Brujeni G.N, Abolhassani M (2020). *Pasteurella multocida* vaccine candidates:A systematic review. Avicenna J. Med. Biotechnol.

[43] Schunicht O.C, Booker C.W, Jim G.K, Guichon P.T, Wildman B.K, Hill B.W (2003). Comparison of a multivalent viral vaccine program versus a univalent viral vaccine program on animal health, feedlot performance, and carcass characteristics of feedlot calves. Can. Vet. J.

[44] Gaudino M, Nagamine B, Ducatez M.F, Meyer G (2022). Understanding the mechanisms of viral and bacterial coinfections in bovine respiratory disease:A comprehensive literature review of experimental evidence. Vet. Res.

[45] Kureljušić B, Weissenbacher-Lang C, Nedorost N, Stixenberger D, Weissenböck H (2016). Association between Pneumocystis spp. and coinfections with *Bordetella bronchiseptica*, *Mycoplasma hyopneumoniae* and *Pasteurella multocida* in austrian pigs with pneumonia. Vet. J.

[46] Croville G, Foret C, Heuillard P, Senet A, Delpont M, Mouahid M, Guerin J (2018). Disclosing respiratory coinfections:A broad-range panel assay for avian respiratory pathogens on a nanofluidic PCR platform. Avian Pathol.

[47] Rawat N, Gilhare V.R, Kushwaha K.K, Hattimare D.D, Khan F.F, Shende R.K, Jolhe D.K (2019). Isolation and molecular characterization of *Mannheimia haemolytica* and *Pasteurella multocida* associated with pneumonia of goats in Chhattisgarh. Vet. World.

[48] Godfray H.C.J, Beddington J.R, Crute I.R, Haddad L, Lawrence D, Muir J.F (2010). Food security:The challenge of feeding 9 billion people. Science.

[49] Griffin D, Chengappa M.M, Kuszak J, McVey D.S (2010). Bacterial pathogens of the bovine respiratory disease complex. Vet. Clin. North Am. Food Anim. Pract..

[50] Bell R.L, Turkington H.L, Cosby S.L (2021). The bacterial and viral agents of BRDC:Immune evasion and vaccine developments. Vaccines (Basel).

[51] Murray G.M, More S.J, Sammin D, Casey M.J, McElroy M.C, O'Neill R.G, Cassidy J.P (2017). Pathogens, patterns of pneumonia, and epidemiologic risk factors associated with respiratory disease in recently weaned cattle in Ireland. J. Vet. Diagn. Invest.

[52] Mistry S.J, Bhanderi B.B, Roy A, Mathakiya R.A, Jhala M.K (2021). Multilocus sequence typing of *Pasturella multocida* isolates from different animal species. Indian J. Vet. Sci. Biotech.

[53] Sakmanoğlu A, Uslu A, Sayin Z, Karyeyen Y, Gölen G.S, İlban A (2021). Investigation of the nontypical *Pasteurella multocida* strains obtained from multiple sources, regions, and times:An unexpected increase was detected. Turk. J. Vet. Anim. Sci.

[54] Liu S, Lin L, Yang H, Wu W, Guo L, Zhang Y, Wang F, Wang X, Song W, Hua L, Liang W, Tang X, Chen H, Peng Z, Wu B (2021). *Pasteurella multocida* capsular:Lipopolysaccharide types D:L6 and A:L3 remain to be the main epidemic genotypes of pigs in China. Anim. Dis.

[55] Kim J, Kim J.W, Oh S.I, So B, Kim W.I, Kim H.Y (2019). Characterisation of *Pasteurella multocida* isolates from pigs with pneumonia in Korea. BMC Vet. Res.

[56] Mahrous E.H, Abd Al-Azeem M.W, Wasel F.A, Younis W (2022). Molecular detection and characterization of *Pasteurella multocida* isolated from rabbits. J. Anim. Health Prod.

[57] Rímac R, Luna L, Hurtado R, Rosadio R, Maturrano L (2017). Detection and genetic characterization of *Pasteurella multocida* from alpaca (*Vicugna pacos*) pneumonia cases. Trop. Anim. Health Prod.

[58] Abd-Elsadek E.L, Mostafa A, Abouelkhair A (2021). Molecular studies on *Pasteurella multocida* in ducks. J. Curr. Vet. Res.

[59] Alonso M.E, González-Montaña J.R, Lomillos J.M (2020). Consumers'concerns and perceptions of farm animal welfare. Animals (Basel).

[60] Bessone F.A, Pérez M.L.S, Zielinski G, Dibarbora M, Conde M.B, Cappuccio J, Alustiza F (2019). Characterization and comparison of strains of *Pasteurella multocida* associated with cases of progressive atrophic rhinitis and porcine pneumonia in Argentina. Vet. World.

[61] Ahmed G, Qadeer M.A, Odugbo M.O, Odita C.I, Makanju O.A, Najeem A.O (2016). Haemorrhagic septicaemia outbreaks in cattle with high mortality following wrong vaccinations in Adamawa and Taraba States, Nigeria. Int. J. Livest. Prod.

[62] Prajapati A, Yogisharadhya R, Mohanty N.N, Kumar S.K, Niszamuddin A, Chanda M.M, Shivachandra S.B (2022). Comparative genome analysis of *Pasteurella multocida* serogroup B:2 strains causing haemorrhagic septicemia (HS) in bovines. Gene.

[63] Gutiérrez A.H, Spero D.M, Gay C, Zimic M, De Groot A.S (2012). New vaccines needed for pathogens infecting animals and humans:One Health. Hum. Vaccin. Immunother.

[64] Liu Q, Hu Y, Li P, Kong Q (2019). Identification of fur in *Pasteurella multocida* and the potential of its mutant as an attenuated live vaccine. Front. Vet. Sci..

[65] Zhao X, Liu Q, Xiao K (2016). Identification of the *crp* gene in avian *Pasteurella multocida* and evaluation of the effects of *crp* deletion on its phenotype, virulence and immunogenicity. BMC Microbiol.

[66] Zhao X, Shen H, Liang S (2021). The lipopolysaccharide outer core transferase genes pcgD and hptE contribute differently to the virulence of *Pasteurella multocida* in ducks. Vet. Res.

[67] Azam F.M, Zamri-Saad M, Rahim R.A, Chumnanpuen P, Othman S (2021). Molecular characterisation of the GdhA-derivative of *Pasteurella multocida* B:2. Pertanika J. Trop. Agric. Sci.

[68] Gong Q, Qu N, Niu M.F, Qin C (2016). Evaluation of immunogenicity and protective efficacy of recombinant ptfA of avian *Pasteurella multocida*. Iran. J. Vet. Res.

[69] Muangthai K, Tankaew P, Varinrak T, Uthi R, Rojanasthien S, Sawada T, Sthitmatee N (2018). Intranasal immunization with a recombinant outer membrane protein H based haemorrhagic septicemia vaccine in dairy calves. J. Vet. Med. Sci.

[70] Wei X, Wang Y, Luo R, Qian W, Sizhu S, Zhou H (2017). Identification and characterization of a protective antigen, PlpB of bovine *Pasteurella multocida* strain LZ-PM. Dev. Comp. Immunol.

[71] Mostaan S, Ghasemzadeh A, Karam M.R.A, Ehsani P, Sardari S, Shokrgozar M.A (2021). *Pasteurella multocida* PlpE protein polytope as a potential subunit vaccine candidate. Vector Borne Zoonotic Dis.

[72] Muenthaisong A, Nambooppha B, Rittipornlertrak A, Tankaew P, Varinrak T, Muangthai K (2020). An intranasal vaccination with a recombinant outer membrane protein H against haemorrhagic septicemia in swamp buffaloes. Vet. Med. Int.

[73] Shivachandra S.B, Yogisharadhya R, Kumar A, Mohanty N.N, Nagaleekar V.K (2015). Recombinant transferrin binding protein A (rTbpA) fragments of *Pasteurella multocida* serogroup B:2 provide variable protection following homologous challenge in the mouse model. Res. Vet. Sci.

[74] Shivachandra S.B, Kumar A, Mohanty N.N, Yogisharadhya R (2017). Immunogenicity of recombinant Omp16 protein of *Pasteurella multocida* B:2 in the mouse model. Indian J. Anim. Sci.

[75] Wu M.C, Lo Y.T, Wu H.C, Wang H.Y, Chu C.Y (2021). Cross-protection of recombinant *Pasteurella multocida* toxin proteins against atrophic rhinitis in mice. Res. Vet. Sci..

[76] Chelliah S, Velappan R.D, Lim K.T, Swee C.W.K, Rashid N.N, Rothan H.A (2020). Potential DNA vaccine for haemorrhagic septicaemia disease. Mol. Biotechnol.

[77] Gong Q, Kong L.Y, Niu M.F, Qin C.L, Yang Y, Li X (2018). Construction of a ptfA chitosan nanoparticle DNA vaccine against *Pasteurella multocida* and the immune response in chickens. Vet. J.

[78] Kang T.L, Chelliah S, Velappan R.D, Kabir N, Mohamad J, Rashid N.N, Ismail S (2019). Intranasal inoculation of recombinant DNA vaccine ABA392 against haemorrhagic septicaemia disease. Lett. Appl. Microbiol.

[79] Yassein A.A.M, Teleb A.A, Hassan G.M, El Fiky Z.A (2021). The immune response and protective efficacy of a potential DNA vaccine against virulent *Pasteurella multocida*. J. Genet. Eng. Biotechnol.

[80] Pegu H, Tamuly S, Sharma R.K (2022). Inmunopotential of *Pasteurella multocida* bivalent outer membrane protein-based vaccine entrapped in aluminum hydroxide nanoparticles. Braz. J. Microbiol.

[81] Rahman M.H, Shahiduzzaman A.N.M, Haque M.E, Nazir K.H.M (2016). Development of experimental oil-based inactivated HS vaccine from field isolates of *Pasteurella multocida* from cattle in Bangladesh. Int. J. Vaccines Vaccin.

[82] Ghadimipour R, Ghorbanpoor M, Gharibi D, Mayahi M (2021). Effects of selected adjuvants on immunogenicity and protectivity of *Pasteurella multocida* bacterin vaccine in chickens. Arch. Razi Inst.

[83] El-Jakee J.K, Moussa I.M, Omran M.S, Ahmed B.M, Elgamal M.A, Hemeg H.A (2020). A novel bivalent Pasteurellosis-RHD vaccine candidate adjuvanted with Montanide ISA70 protects rabbits from lethal challenge. Saudi J. Biol. Sci.

[84] Domínguez-Odio A, Pérez O, Batista-Duharte A, Cala-Delgado D (2022). Technology surveillance in veterinary vaccine adjuvants (2015–2022):University-industry interaction. J. Pharm. Pharmacogn. Res.

[85] Homayoon M, Tahamtan Y, Kargar M, Hosseini S.M.H, Sepahy A.A (2018). *Pasteurella multocida* inactivated with ferric chloride and adjuvanted with bacterial DNA is a potent and efficacious vaccine in Balb/c mice. J. Med. Microbiol.

[86] Tanwar H, Yadav A.P, Singh S.B, Ganju L (2016). Immunity against *Pasteurella multocida* in animals vaccinated with inactivated *Pasteurella multocida* and herbal adjuvant 'DIP-HIP'. J. Vaccines Immunol.

[87] Gong Q, Peng Y.G, Niu M.F, Qin C.L (2020). Research note:The immune enhancement ability of inulin on ptfA gene DNA vaccine of avian *Pasteurella multocida*. Poult. Sci.

